# Chloral Hydrate, Locally

**Published:** 1886-10

**Authors:** 


CHLORAL HYDRATE LOCALLY.—The local application of chloral hydrate is very servicable in many diseases, both on account of the relief of pain afforded and the cleansing of the parts. For cancerous ulceration of glands and of the uterus, phagadenic ulcerations, eczema, impetigo, ulcerated legs, herpes zoster, pleurodynia and neuralgia, local employment of chloral, half an ounce in a pint of water, with a little glycerine, has been productive of much benefit. Ulcerated surfaces become healty by comparsion, discharges less offensive and pain is reduced to a minimum. These results are probably due to a direct action on the peripheral nerve terminations. —*Med. World.*

